# Effects of sub-lethal concentrations of copper ammonium acetate, pyrethrins and atrazine on the response of *Escherichia coli* to antibiotics

**DOI:** 10.12688/f1000research.17652.1

**Published:** 2019-01-09

**Authors:** Hyunwoo Jun, Brigitta Kurenbach, Jack Aitken, Alibe Wasa, Mitja N.P. Remus-Emsermann, William Godsoe, Jack A. Heinemann

**Affiliations:** 1 School of Biological Sciences, University of Canterbury, Christchurch, 8140, New Zealand; 2 Centre for Integrated Research in Biosafety and Centre for Integrative Ecology, University of Canterbury, Christchurch, 8140, New Zealand; 3 Biomolecular Interaction Centre, University of Canterbury, Christchurch, 8140, New Zealand; 4 Bio-Protection Centre, Lincoln University, Lincoln, 7647, New Zealand

**Keywords:** biocides, antibiotic resistant bacteria, antibiotics, copper, pyrethrins, atrazine

## Abstract

**Background:** Antibiotic resistance in human and animal pathogens is mainly the outcome of human use of antibiotics. However, bacteria are also exposed to thousands of other antimicrobial agents. Increasingly those exposures are being investigated as co-selective agents behind the rapid rise and spread of resistance in bacterial pathogens of people and our domesticated animals.

**Methods:** We measured the sub-lethal effects on antibiotic tolerance of the human pathogen/commensal *Escherichia coli* caused by exposure to three common biocide formulations based on either copper, pyrethrins, or atrazine as active ingredients. The influence of the efflux pump AcrAB-TolC was investigated using deletion strains, and the persistence of observed effects was determined.

**Results:** Some effects were seen for all biocides, but the largest effects were observed with copper in combination with the antibiotic tetracycline. The effect was caused by both the induction of the adaptive efflux system and by chelation of the antibiotic by copper. Finally, persistence of the adaptive response was measured and found to persist for about two generations.

**Conclusions:** Through a combination of microbe-chemical and chemical-chemical interactions, humanity may be creating micro-environments in which resistance evolution is accelerated.

## Introduction

Besides antibiotics, a growing number of anthropogenic products are being found to affect antibiotic resistance in microorganisms ( Heinemann & Kurenbach, 2017; Knöppel *et al.*, 2017; Molina-González *et al.*, 2014). These include non-antibiotic therapeutics ( Kristiansen, 1992; Maier *et al.*, 2018), food sweeteners ( Wang *et al.*, 2018), food perservatives ( Capita & Alonso-Calleja, 2013; Capita *et al.*, 2014), emulsifiers used in food and medicine ( Kurenbach *et al.*, 2017), paints, and cleaning products ( Buffet-Bataillon *et al.*, 2016; Molina-González *et al.*, 2014).

The world’s industrial capacity to produce, distribute and consume manufactured chemical products is at an all time high and growing ( American Chemistry Council, 2016). In the United States alone, 13,000 kg of industrial chemicals are produced per capita per year, and over 11,000 kg of 8,000 chemicals are produced or imported per capita per year ( Wang *et al.*, 2018).

Manufactured chemicals contribute to pollution, which is the leading cause of disease and premature death worldwide ( Landrigan *et al.*, 2018). The Lancet Commission on Pollution and Health said that less than half of “high-production volume chemicals have undergone any testing for safety or toxicity, and rigorous pre-market evaluation of new chemicals has become mandatory in only the past decade and in only a few high income countries. The result is that chemicals and biocides whose effects on human health and the environment were never examined have repeatedly been responsible for episodes of disease, death, and environmental degradation” ( Landrigan *et al.*, 2018).

To our knowledge, pre-market assessments of biocides that include tests of sub-lethal effects on microorganisms have not been performed yet ( Kurenbach *et al.*, 2015), although this may be changing, at least in Europe ( Buffet-Bataillon *et al.*, 2016). For every human exposure to a biocide, there may be 10s of trillions of exposures in our personal microbiota, not to mention microbiota exposures in soil, water and air, and on plants, livestock, companion animals and insects ( Claus *et al.*, 2016; Imfeld & Vuilleumier, 2012; Motta *et al.*, 2018).

We have previously shown that active ingredients and commercial formulations based on dicamba, glyphosate, and 2,4-D induced changes in the response of *Escherichia coli* and *Salmonella enterica* to five different antibiotics from different classes. Increases in tolerance to antibiotics could be attributed in part to increased production of efflux pumps from the resistance-nodulation-division (RND) family ( Kurenbach *et al.*, 2015; Kurenbach *et al.*, 2017). Unfortunately, the diversity of active and adjuvant ingredients of the tested herbicides provide little basis to produce general predictions of effects on different bacteria because of a common chemistry. Thus, at present, products must be tested on a case-by-case basis to determine whether or not there are sub-lethal responses in bacteria of interest.

The aim of the work described here was to determine whether other biocides used in agriculture and urban environments could induce a similar response in *E. coli*. The biocides used in the experiments were commercial formulations of a fungicide (copper ammonium acetate), an insecticide (pyrethrins) and an herbicide (atrazine).

We measured the initial response of bacteria to chemical exposures by the adaptive changes in the expression of TolC, an efflux pump component that controls transport across membranes ( Corona & Martinez, 2013). This response is reversible in time, but may be heritable through epigenetic transmission ( Bootsma *et al.*, 2012; Motta *et al.*, 2015). We used one biocide-antibiotic combination to attempt to empirically measure the transgenerational longevity of the adaptive response.

## Methods

### Strains and chemicals

Strains used in this study are detailed in Table 1. Liquid cultures were grown in LB Lennox (Invitrogen, Auckland, NZ) at 37°C in a rotary incubator. Antibiotics used were tetracycline (Tet, Sigma, Auckland, NZ), streptomycin (Str, Sigma, Auckland, NZ), kanamycin (Kan, Gibco, Auckland, NZ), and ciprofloxacin (Cip, Pentex, Auckland, NZ). Biocides were commercial formulations Yates Liquid Copper Fungicide (Yates, Auckland, NZ) containing 92.8 g/L of Copper (Cu ^2+^) in the form of copper ammonium acetate, Pyrethrum Natural Insect Spray (Yates, Auckland, NZ) containing pyrethrins (14 g/L) and 56.5 g/L of piperonyl butoxide, and Atranex WG (Adama, Nelson, NZ), containing 900g/kg atrazine. Relevant concentrations are given in the main text or Figure legends.

**Table 1.** *E. coli* strains used in this study.

*E. coli* strain	Genotype/relevant characteristic	Reference/Source
BW25113	Wild type. F-, λ-, Δ( *araD*- *araB*)567, Δ *lacZ4787*(:: *rrnB*-3), *rph-1,* Δ( *rhaD-rhaB*)568, *hsdR*514	( Baba *et al.*, 2006)
CR7000	BW25113 Δ *acr*A	( Ruiz & Levy, 2014)
CR5000	BW25113 Δ *acr*B	( Ruiz & Levy, 2014)
JW5503	BW25113 Δ *tol*C:: *kan,* Kan ^R^	( Baba *et al.*, 2006)
BW25113 (pHJ01)	BW25113 pHJ01 (pFru - P _tolC__mScarlet)	This work

### Minimum inhibitory concentration (MIC) and antibiotic response

Antibiotic responses were determined as described previously ( Kurenbach *et al.*, 2015). In brief, *E. coli* was grown to saturation (ca. 2 x 10 ^9^ cfu/mL) in LB, and serial dilutions were plated on LB in the presence of antibiotics and/or biocides. When added, biocide concentrations were constant, while antibiotic concentrations varied. Plates were incubated at 37°C and examined daily for up to 10 days, at which point no new colonies emerged. To account for day to day variability, cfu counts were normalised to growth on nonselective medium. The efficiency of plating (EoP) is the ratio of a culture’s titre (cfu/mL) on treatment plates to the titre on LB [(cfu/mL) _treatment_/ (cfu/mL) _LB_] ( Rosner, 1985). The detection range was an EoP of ca. 1 to 10 ^-7^.

### Dose response

The concentration of biocide that caused a significantly different response to an antibiotic (“dose response”) was determined as described previously ( Kurenbach *et al.*, 2015). In brief, *E. coli* were grown to saturation in LB and a serial dilution was plated on LB agar plates supplemented with varying concentrations of biocide and a constant concentration of antibiotic. The antibiotic concentration used was the one causing the greatest difference in EoP in the antibiotic response experiments. The inducing concentration of a biocide was defined as the lowest concentration for which a change occurred that was a) statistically significant and b) showed an at least 100-fold difference in EoP compared to the control containing only antibiotic. Plates were incubated at 37°C and examined daily for up to 10 days, at which point no new colonies emerged.

### Plasmid construction

To construct plasmid pHJ01, *E. coli* BW25113 (GenBank accession number CP009273) was used as a template for the 204 bp upstream of the start codon of *tolC*. The *tolC* promoter was amplified by PCR and fused to *mScarlet-I* which was amplified from pTriEx-RhoA-wt_mScarlet-I_SGFP2 (Addgene plasmid #85071) and *Hin*dIII digested pFru97 ( Tecon & Leveau, 2012) by isothermal assembly ( Gibson *et al.*, 2009; Schlechter *et al.*, 2018). Touchdown PCRs were performed as described previously ( Schlechter *et al.*, 2018) using Phusion High-Fidelity DNA polymerase (Thermo Scientific, Auckland, NZ). Primers used were FWD_TolC (5' **CAG GAC GCC CGC CAT AAA CTG CCA GGA ATT GGG GAT CGG A**TG TTA ATG TCC TGG CAC TAA TAG TGA ATT AAA TGT 3’; Tm: 60°C), REV_TolC (5' TCG CCC TTG CTC ACC ATG GT *T TGC ATT CCT TGT GGT GAA GCA G* 3'; Tm: 60°C), TolC_mScarlet_FWD (5' *CTT CAC CAC AAG GAA TGC AA*A CCA TGG TGA GCA AGG GC 3’; Tm: 70°C), and mScarlet_REV (5' **TTA CTG GAT CTA TCA ACA GGA GTC CAA GCT CAG CTA ATT** ACT TGT ACA GCT CGT CCA TGC 3'; Tm: 71°C), where nucleotides shown in bold font are complementarity to the vector, and nucleotides shown in italics overlap with other primers. pHJ01 transformands of BW25113 were selected on kanamycin.

### Microscopy

Prior to microscopy, cells grown for 180 min either in LB or in LB + 450 µg/mL copper were fixed using 4% paraformaldehyde as described previously and stored at -20°C in 1:1 ethanol:phosphate buffered saline ( Akkermans *et al.*, 1996; Kowalchuk *et al.*, 2004). Fixed cells were examined with an Axio Imager. M1 (Zeiss, Oberkochen, Germany) using an EC Plan-Neofluar 100x objective (NA 1.30) and Zeiss filter set 43HE (BP 550/25 (HE); FT 570 (HE); BP 605/70 (HE)). Multichannel images were acquired using an AxioCam 506 mono camera (Zeiss) in differential interference contrast (DIC) and Zeiss filter set 43HE. Single cell fluorescence was determined as described previously ( Remus-Emsermann *et al.*, 2016).

### Tetracycline chelation

Seven Erlenmeyer flasks (50 mL) containing LB (10 mL) were supplemented with copper (450 µg/mL) and tetracycline (35 μg/mL) (Flasks 1–4), tetracycline (35 μg/mL) without copper (Flasks 5 and 6), or copper (450 µg/mL) without tetracycline (Flask 7) at t _0_. All flasks were incubated continuously at 37°C with aeration. *E. coli* BW25113 was grown to saturation without selection and approximately 10 ^4^ cfu were used to inoculate flasks 1, 6 and 7 at t _0_, and flasks 2 and 3 at t _24_ and t _48_, respectively. Flasks 4 and 5 were inoculated at t _96_. The culture in each flask was monitored for growth every 24 hours by plating appropriate dilutions onto LB agar plates.

### Measuring the persistence of copper-induced tetracycline resistance

*E. coli* was grown to saturation with aeration at 37°C in liquid LB medium supplemented with both copper (450 µg/mL) and tetracycline (15 µg/mL) for 3 days. This culture was diluted 100-fold into 10 mL LB medium supplemented with only tetracycline (15 µg/mL) and incubated at 37°C for 12 hours with aeration. The concentration of *E. coli* at the start and end of the experiment was determined using a haemocytometer.

### Statistical analysis

R (version 3.2.0) was used for all statistical analyses ( R Core Team, 2013). In experiments testing the responses to antibiotics during exposure to biocides we were interested in effects on EoP that were different in antibiotic+biocide combinations compared to either substance in isolation. We therefore tested the log-transformed EoP scores using a multifactor analysis of variance (ANOVA) by evaluating the significance of the antibiotic by biocide interaction term. Antibiotic concentrations were treated as separate categories in the ANOVA. Plots of residuals were used to test for violations of assumptions. We fit these models using the lm function.

Since many data points used for the determination of the concentration of biocide that caused a significantly different response to an antibiotic were near or below the detection limit, residuals from a standard ANOVA were not normally distributed. We therefore used the equivalent non-parametric Kruskal-Wallis one-way ANOVA to test for differences in log-transformed EoP/EoP _(0)_ scores among biocide concentrations. The P-value reported is derived for a null model where EoP/EoP _(0)_ is the same across all biocide concentrations versus and alternative model where the ratio differs among some concentrations.

We tested whether cfu scores depended on “flask” using a single factor ANOVA at 24 and 48 hours post inoculation. In each case, we first used an analysis of covariance (ANCOVA) to test if cfu scores post inoculation were confounded with the cfu count and the time of inoculation. Cfu scores at inoculation did not influence final scores (data not shown). Cfu scores were transformed to log (cfu +0.0001) to ensure normality of the residuals. We used a sequential Bonferroni contrast to test for differences among treatments (Flasks 1–4) and between treatments and controls (flasks 1 and 6, 1 and 6, and 4 and 5). Residuals were used to check assumptions. With the exception of two low-influence outliers, the data matched our expectations under normality.

Where fluorescence was measured, differences between median fluorescence values of the reporter strain grown under two conditions (+/- copper) were determined using a non-parametric Mann-Whitney U T-test because residuals were not normally distributed. Violin plots were created using ggplot2 ( Wickham, 2016).

## Results

### Effects of biocides on antibiotic response

MIC was defined as the minimum concentration of agent in an agar plate at which no growth was observed after ca. 10 ^8^ cfu were applied to the surface. It was not possible to determine the MIC for atrazine because *E. coli* BW25113 survived to the limit of solubility of atrazine in our standard culture medium, LB. The No Observable Effect Level (NOEL) was defined as the highest concentration of a substance that had no effect on the EoP ( Table 2).

**Table 2.** Relevant biocide NOEL and MIC values.

*E. coli* strain	Biocide	NOEL (µg/ml)	MIC (µg/ml)
BW25113 (WT)	Copper	450	1635
	Pyrethrins	140	2300
	Atrzine	1000	> 2000
CR7000 (Δ *acr*A)	Copper	450	1430
CR5000 (Δ *acr*B)	Copper	450	1230
JW5503 (Δ *tol*C)	Copper	450	700

**Figure 1.**
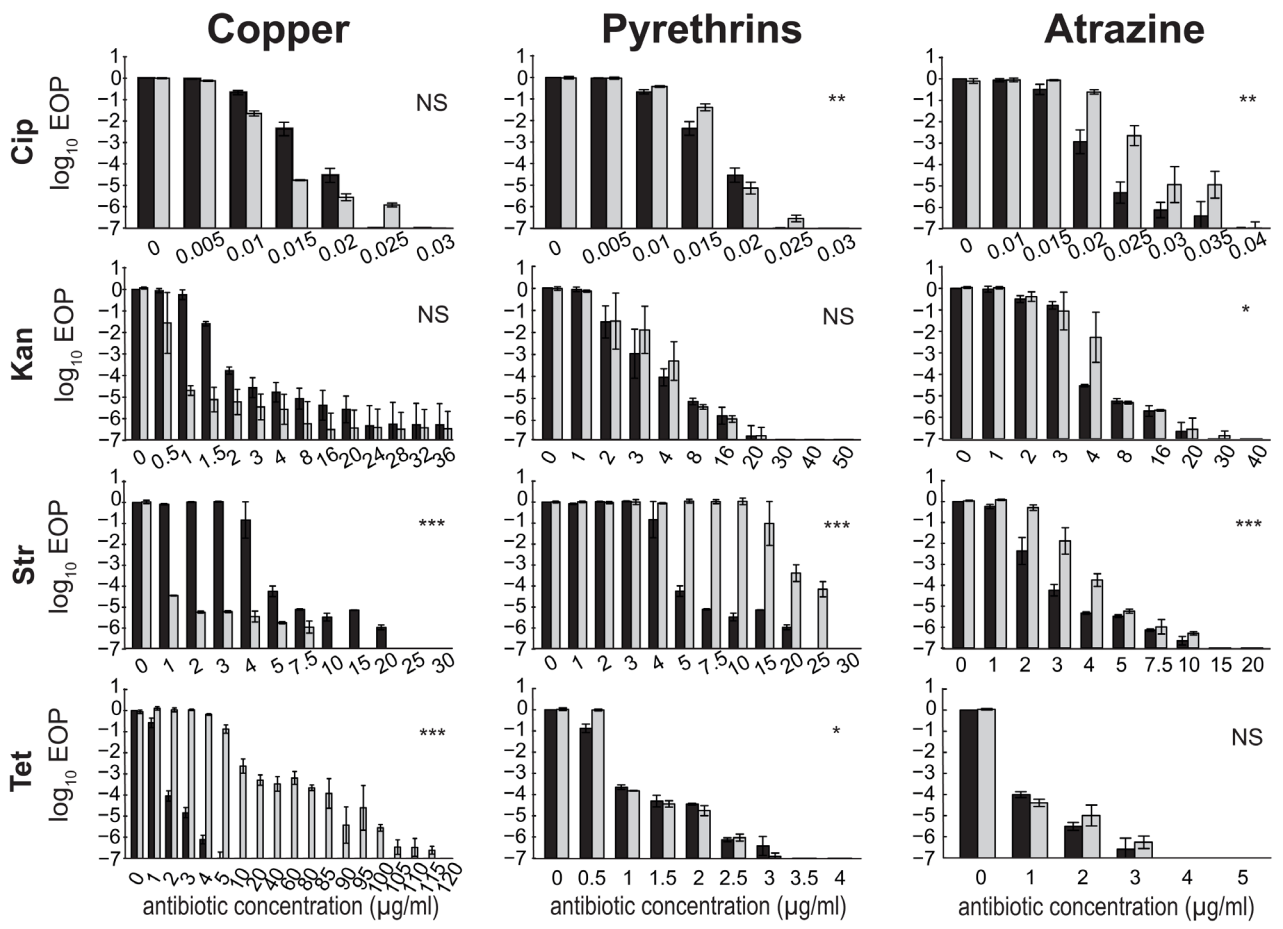
Change in EoP when *E. coli* BW25113 is (grey) or is not (black) exposed to biocides. The x-axes scale is antibiotic concentrations in µg/mL. Biocide concentrations used were 450 µg/mL for copper ammonium acetate, 140 µg/mL for pyrethrin, and 1000 µg/mL for atrazine. Values are means of at least three independent experiments; error bars are standard errors (SEM, with SEM=standard deviation/√n). Asterisks indicate P-values for antibiotic*herbicide interaction terms (see Materials and Methods). * *P*<0.05; ** *P*<0.01; *** *P*<0.001; ns, not significant.

Bacteria were cultured on LB agar supplemented with one of the three commercial formulations of biocide (at respective NOEL concentrations) as well as different concentrations of selected antibiotics. Changes in response to particular concentrations of antibiotic because of exposure to the biocide are revealed as a differential EoP ( Figure 1). As reported for other biocide*antibiotic combinations, the observed responses were specific for the combination of biocide and antibiotic used ( Kurenbach *et al.*, 2015; Kurenbach *et al.*, 2017). We observed increases and decreases in tolerance to different antibiotics as well as no effect in some cases. As a conservative threshold, we used the antibiotic concentration for which we saw an at least 10 ^3^-fold decrease in EoP as the cut-off point to determine the fold-change in survival ( Table 3).

**Table 3.** Fold change shift in antibiotic effectiveness following exposure to biocides.

	Copper	Pyrethrins	Atrazine
Cip	1.3 ^b^	0 ^a^	1.2
Kan	2 ^b^	2 ^b^	2
Str	5	5	1.3
Tet	40	0 ^a^	0

^a^While EoP dropped below our threshold at the same concentration in the presence and absence of biocides, the ANOVA showed a statistically significant interaction term for this combination.

^b^The ANOVA did not show a statistically significant interaction term, despite the drop in EoP below our threshold at different concentrations.

Copper significantly increased the EoP over a 40-fold concentration range of tetracycline (from 2 to 80 µg/mL) and decreased it on a 5-fold concentration range of streptomycin (from 5 to 1 µg/mL). Copper caused non-statistically significant decreases in the EoP on either ciprofloxacin or kanamycin.

Pyrethrins increased EoP over a 5-fold streptomycin concentration range. They caused a statistically significant difference in EoP on ciprofloxacin, but no change in MIC. This has been sometimes observed for various herbicide-antibiotic combinations ( Kurenbach *et al.*, 2015; Kurenbach *et al.*, 2018).

Atrazine caused statistically significant but small increases in EoP on ciprofloxacin, kanamycin, and streptomycin.

### Determining the minimum biocide concentration causing a change in the response to antibiotics

In the experiments described above, the concentration of antibiotic was varied while the biocide concentration was constant. To determine the minimum biocide concentration necessary to cause the observed effects, we chose an antibiotic concentration for which there was a maximum resolution between treatments and decreased the biocide concentration for each biocide.

**Figure 2.**
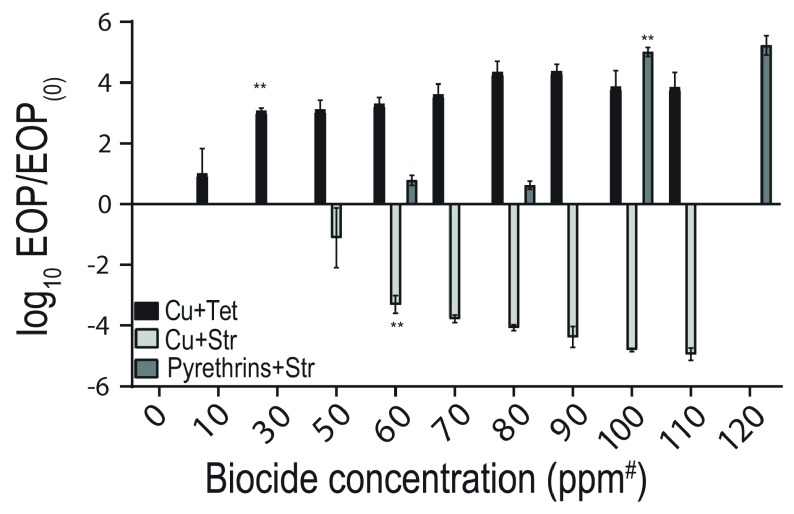
Dose response curves for *E. coli* BW25113. Antibiotic concentrations used (in µg/mL) were as follows: Tet: 10 µg/mL for copper; Str: 2 µg/mL for copper and 10 µg/mL for pyrethrins. Values are means of at least 3 independent experiments; error bars are standard errors (SEM, with SEM=standard deviation/√n). Asterisks indicate the lowest biocide concentration for which a statistically significant change in EoP by at least 100-fold compared to the antibiotic only occurred. * *P*<0.05; ** *P*<0.01; *** *P*<0.001; ns, not significant.

As a conservative measure, we report the biocide concentration that caused a statistically significant and at least a 100-fold change in the EoP compared to the EoP of the antibiotic-only plate (EoP _(0)_). To aid visualization, we calculated log EoP/EoP _(0)_ ( Figure 2). A value >0 indicates that the biocide increases EoP of bacteria on higher concentrations of the antibiotic. Our threshold of a 100-fold change was reached at 120 µg/mL copper with tetracycline, 250 µg/mL copper with streptomycin, and 100 µg/mL pyrethrins with streptomycin.

The minimum biocide concentration was not determined for some other statistically significant combinations shown in Figure 1. This was because the affected antibiotic concentrations were of such a small range. As a consequence, we also concentrated on copper exposures in the remainder of the study.

### Reversibility of phenotype as evidence of an adaptive response

We previously found that the herbicidal formulations based on 2,4-D, dicamba and glyphosate ( Kurenbach *et al.*, 2015), as well as the corresponding purified active ingredients ( Kurenbach *et al.*, 2017) caused changes in the expression pattern of genes that may alter antibiotic susceptibility. This response is phenotypic, resulting from a change in gene expression rather than genotype. It is distinguished from the outgrowth of rare spontaneous mutants by the uniform reversion on the population level when the environment changes ( Motta *et al.*, 2015).

We further characterized the copper-induced tetracycline response as an adaptive response by following the phenotype of induced clones. Randomly chosen colonies from cultures plated on LB, LB+Tet, or LB+Tet+Cu were transferred to plates containing 35 μg/mL tetracycline or LB. At this tetracycline concentration, *E. coli* survived only when simultaneously exposed to copper.

Regardless of whether the colonies were transferred from LB or LB+Tet+Cu, they all again formed colonies on LB. However, none of the colonies transferred from either LB or LB+Tet+Cu grew on LB+Tet plates, indicating that the response to tetracycline was reversible and dependent upon ongoing stimulation by copper.

### Dependence on the AcrAB-TolC efflux pump as evidence of an adaptive response

**Figure 3.**
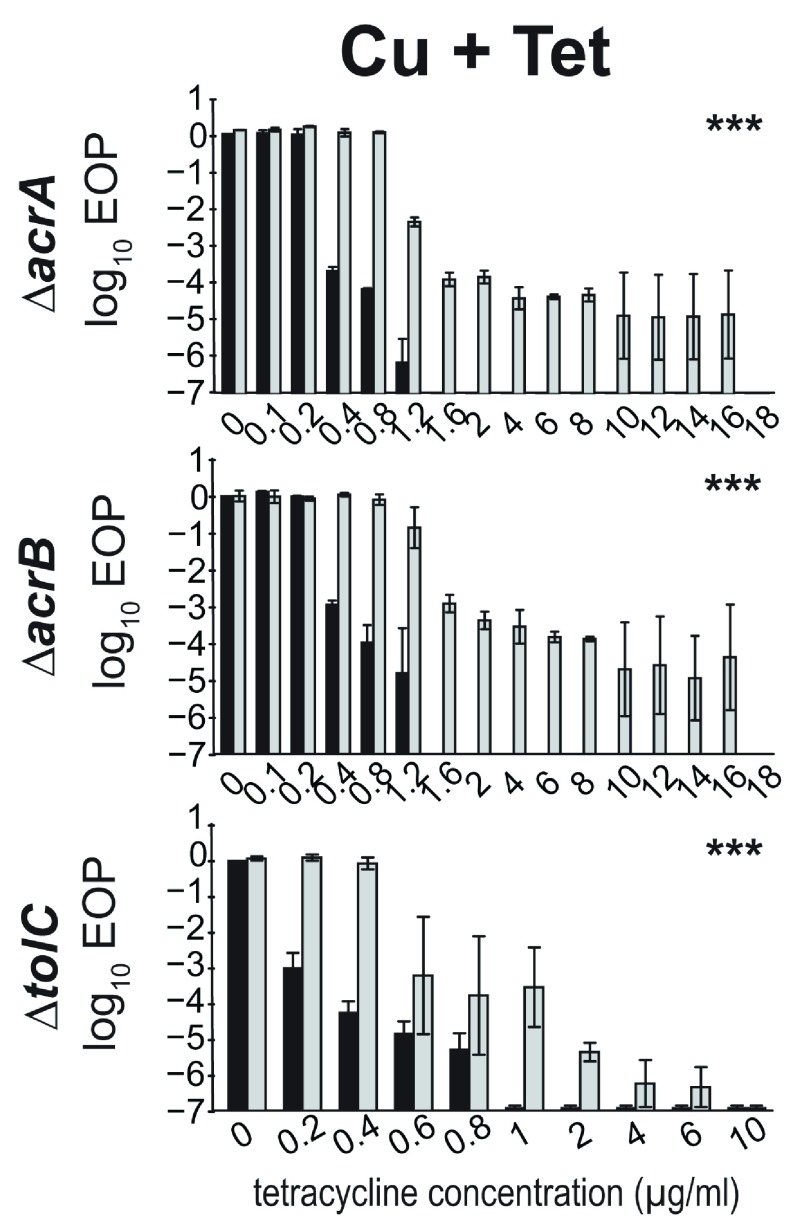
Change in EoP when *E. coli* deletion strains are (grey) and are not (black) exposed to Cu in the presence of Tetracycline. The x-axis indicates antibiotic concentration in µg/mL. Copper was added at 450 µg/mL. Error bars are standard errors (SEM, with SEM=standard deviation/√n). Asterisks indicate *P* values for interaction terms (see Materials and Methods). * *P*<0.05; ** *P*<0.01; *** *P*<0.001; ns, not significant.

The efflux pump AcrAB-TolC was shown to contribute to the altered EoP of *E. coli* on different antibiotics when simultaneously exposed to various herbicides ( Kurenbach *et al.*, 2017). Here, the same set of strains from an isogenic series carrying single gene deletions, Δ *acr*A *,* Δ *acr*B and Δ *tol*C, were used to test whether copper induced an adaptive response via this pump. NOEL and MIC of copper were determined for all three strains ( Table 2). Changes in EoP on tetracycline-supplemented media were measured as described above, using the NOEL copper concentration ( Figure 3).

The MIC but not the NOEL of tetracycline was lower in all three deletion strains compared to the wildtype BW25113. This is consistent with the observations of others ( de Cristóbal *et al.*, 2006) and suggests that the AcrAB-TolC efflux pump is responding to copper and contributing to tetracycline resistance. Concurrent copper exposure significantly increased tolerance to tetracycline in all strains. However, with increases of 4-fold for Δ *acr*A, 22.5-fold for Δ *acr*B, and 5-fold for Δ *tol*C these effects were smaller than those observed for the parental strain (40-fold). This suggests that the AcrAB-TolC efflux pump is responding to copper and contributing to tetracycline resistance, but it is not the only mechanism involved.

### TolC was induced by copper

Accumulation of copper directly effects the transcription factor MarR, derepressing the MarRAB operon ( Hao *et al.*, 2014). Increased production of the transcription factor MarA leads to increased transcription of among others the *acrAB* and *tolC* genes ( Weston *et al.*, 2018). We chose to investigate this further by using a *tolC* reporter strain.

The *E. coli* strain BW25113 (pHJ01) has the mScarlet fluorescent protein open reading frame transcriptionally fused to the *tolC* promoter region. This technique was used previously to demonstrate e.g. the accessibility of fructose to bacterial cells on leaves and the availability of phenol to bacteria on leaves ( Leveau & Lindow, 2001; Sandhu *et al.*, 2007).

**Figure 4.**
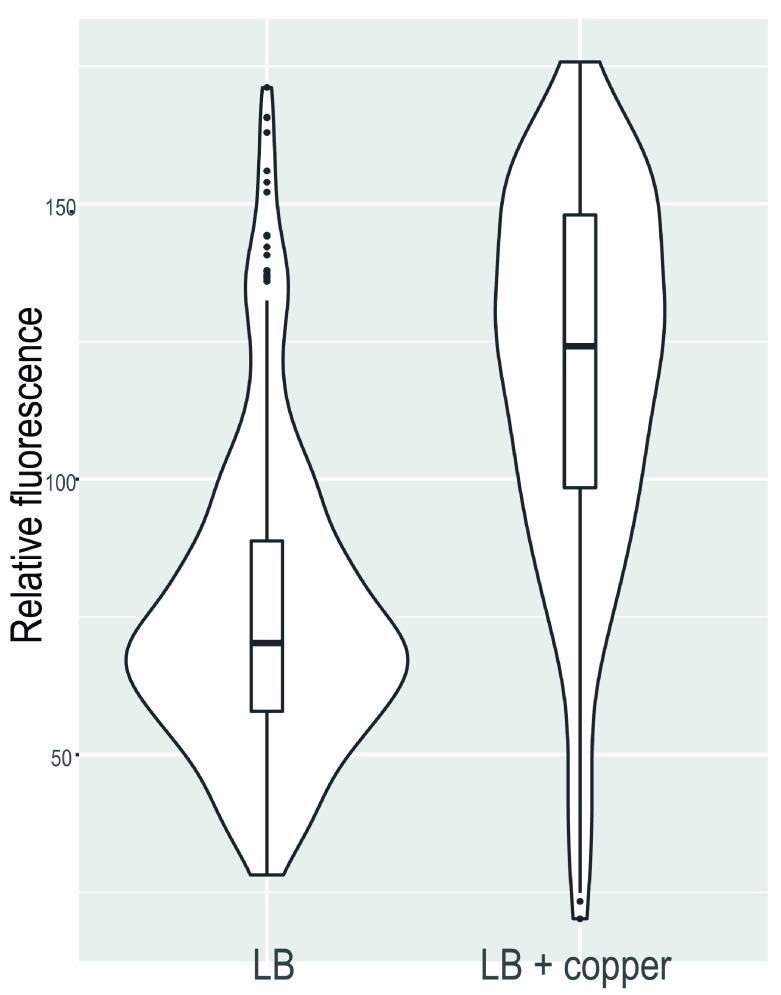
Single-cell fluorescence intensity of *E. coli* BW25113 (pHJ101) expressing red fluorescent mScarlet protein under the control of the tolC promoter without (left) or with (right) copper exposure. The violin plots show the distribution of the single-cell fluorescence within the cell population. The median is depicted by the bar in the box; the box represents the 25% and 75% quartiles.

The fluorescence of BW25113 (pHJ01) was statistically significantly lower (p < 0.001) when cultured in LB compared to LB + 450 µg/mL copper ( Figure 4). The median relative fluorescence of the reporter strain increased 77% from 70 arbitrary fluorescence units (afu) when cultured in LB to 124 afu when cultured with additional copper. This indicates induction of *tolC* by the copper fungicide.

### Copper directly reduced available tetracycline

Copper had a large effect on the EoP of *E. coli* exposed to tetracycline, increasing the concentration necessary to decrease EoP by >10 ^3^-fold from 2 to 80 µg/mL tetracycline. This was the largest effect of any biocide on any antibiotic that we have observed. When cultured in a combination of copper and tetracycline at copper-induced sub-lethal concentrations of tetracycline, we observed a significant delay in the growth of the culture. This could be due to copper chelation of tetracycline ( Tong *et al.*, 2015), or to the outgrowth of rare tetracycline resistant mutants. Since we have not detected the latter (see above), we tested the former hypothesis.

**Figure 5.**
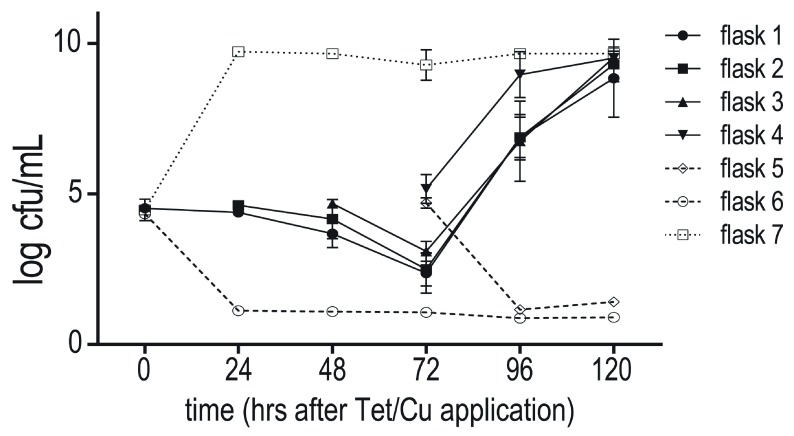
Chelation of tetracycline by copper. X-axis is time in hrs after adding copper and tetracycline (flasks 1–4, solid lines), tetracycline (flasks 5 & 6, dashed lines), or copper (flask 7, dotted line). The first data point in each series indicates time of inoculation with *E. coli* BW25113. Values are means of three independent experiments ± SD.

A series of *E. coli* BW25113 cultures were used to estimate tetracycline bioavailability. The series was composed of four flasks with a medium supplemented with copper and tetracycline and incubated at 37°C with aeration. The medium in the flasks was inoculated with bacteria in successive 24 hour intervals (flask 1 at t _0_ – flask 4 at t _72_) and the titre of each culture was determined at the same intervals by plating dilutions of samples on LB. Control cultures with medium supplemented with only tetracycline were started in parallel with flasks 1 and 4, and a positive control culture using medium only supplemented with copper was inoculated in parallel to flask 1. These controls showed that tetracycline alone, even after 92 hours of pre-incubation, prevented growth of the culture, and that the copper concentration was sub-lethal. Cultures began to grow only after the medium with a mixture of copper and tetracycline was over 72 hours old ( Figure 5).

Using ANOVAs, we tested for significant differences between flasks in cfu counts 24 and 48 hours after inoculation with bacteria (see Underlying data: ‘Chelation experiment_ANOVA tables’; https://doi.org/10.17605/OSF.IO/RZKWU ( Kurenbach, 2018)). At 24 hrs, the cfus of flask 4, inoculated at t _72_, was significantly different to either flasks 1, 2, or 3. At 24 hrs, these flasks had not passed the t _72_ point. At 48 hrs, flasks 3 and 4, now both past t _72_, were not significantly different from each other. Flask 4 was still different from flasks 1 and 2, while differences between 3 and 1 and 2 were not significant or marginally significant, respectively. This is in general agreement with the interpretation that bacteria start growing after t _72_ regardless of the point in time at which they were inoculated (see Underlying data: ‘Chelation experiment_ANOVA tables’; https://doi.org/10.17605/OSF.IO/RZKWU ( Kurenbach, 2018)).

This observation is consistent with the notion that copper forms a complex with tetracycline ( Tong *et al.*, 2015), or facilitates degradation of tetracycline, over time. Because tetracycline is bacteriostatic, the bacteria are able to recover once the effective concentration of tetracycline falls to sub-inhibitory levels.

### Measuring persistence of the adaptive phenotype

*E. coli*’s response to copper exposure was consistent with an adaptive response through a change in efflux pump levels rather than a change in DNA sequence conferring antibiotic resistance. We therefore hypothesized that the tetracycline-resistant physiotypes created by the adaptive response should continue to reproduce in medium supplemented with tetracycline above the MIC until the number of efflux pumps, and possibly other contributing factors, per cell fell below an efficacious threshold ( Bootsma *et al.*, 2012; Motta *et al.*, 2015).

This hypothesis could be tested by determining the number of generations *E. coli* was able to reproduce after removal of copper but not tetracycline from a previously induced (Cu+Tet) culture. A complication was encountered when we observed a reversible filamentation of the bacteria after their transfer from a medium with both copper and tetracycline. *E. coli* are known to form filaments when stressed ( Justice *et al.*, 2006). Filamentation made the determination of growth by measuring OD _600_ inaccurate. To address this, densities of bacteria were determined visually using a haemocytometer.

Immediately after transfer to tetracycline-supplemented medium, the concentration of bacteria was determined. The cultures were then incubated at 37°C for 16 hours. Testing the limits of our method, we were consistently able to distinguish 4-fold differences in population growth, i.e. two generations. Our experimental data fell below that threshold, with populations growing by ca. 3-fold, or just over one generation. We therefore estimate that the adaptive phenotype in this experiment was heritable for less than two generations.

## Discussion

About 2 million metric tons of the 30 most commonly used commercial pesticides are released into the environment annually worldwide. Of these, 55.4% are herbicides, 28.6% are fungicides and 5.7% are insecticides ( Casida & Bryant, 2017). Despite their long and widespread use, to our knowledge they have never been tested for sub-lethal effects on potential human or animal pathogenic bacteria.

We have tested three common pesticides for sub-lethal effects on the bacterium *E. coli*. Copper-based formulations are the third largest fungicide usage group. The triazine herbicide ingredient atrazine is by amount used the third most commonly used herbicide in the world. Pyrethroids are medium use insecticides, occupying positions of 11, 12, 16 and 26 of the top 30 insecticides used worldwide ( Casida & Bryant, 2017).

Similar to our previous findings for the herbicides based on glyphosate, dicamba, and 2,4-d active ingredients, the three biocides tested here did alter the response of the human and animal commensal and potential pathogen *E. coli* to some clinical antibiotics. The concentrations of biocide that caused the change in response to antibiotics were at or below label-recommended application rates, which are 30 - 2320 µg/mL for copper, 70 µg/mL for pyrethrins, and 500 µg/mL for atrazine.

Streptomycin resistance was most affected by pyrethroids, while effects on other antibiotics tested were small. Moreover, results were not statistically significant for the other tested aminoglycoside antibiotic, kanamycin. Likewise, atrazine caused only small effects for all antibiotics tested. We observed the largest changes using copper, which increased survival on 40 times higher concentrations of tetracycline.

The response seen to tetracycline from copper exposure was the largest we have observed from a biocide and antibiotic combination. Some of this is attributed to the chelation of copper by tetracycline, resulting in a decrease in effective concentration of both agents. Nevertheless, some of the response was confirmed to be adaptive because as shown by use of the strains with gene deletions, it depended in part on *acrA, acrB* and *tolC*. Moreover, exposure to copper was directly observed to increase the expression of the red fluorescent protein gene *mScarlet* under the control of the *tolC* promoter, and the fully susceptible phenotype was uniformly restored to the population when induced bacteria were transferred to LB+Tet medium. Because the gene deletion strains continued to respond to copper and tetracycline, the full effect of copper was not explained only through the expression of the AcrAB-TolC efflux pump.

Copper is a common supplement for animal feeds which can also contain traces of copper from biocide residues. In a European Union survey of copper content in animal feed used in member countries, copper was found over a broad range of concentrations (in the mg/kg range) and mean concentrations of 8 to ~20 mg/kg in the feed of most surveyed animals, including pets such as dogs and cats. The highest mean was 119 mg/kg for piglets ( EFSA, 2016). The lowest statistically significant tetracycline-resistance inducing concentration of copper in our study was 120 mg/L, just above routine piglet exposures. Other exposures to copper from use of biocides would be in addition to these.

Animal feed can also be unintentionally contaminated with antibiotics. Tetracycline-class antibiotics are approved for use in animal feed and are among the most frequently used. This alone resulted in concentrations of chlortetracycline and doxycycline at concentrations of 10 mg/kg and 4 mg/kg, respectively, in the feces of pigs. The level was high enough to select for resistance ( Gavilán *et al.*, 2015; Granados-Chinchilla & Rodríguez, 2017).

A study in Vietnam that examined nearly 1500 chicken and pig feed formulations estimated that 77.4 mg and 286.7 mg, respectively, of antimicrobials were used to raise each 1 kg of animal. The level of antimicrobial agent in the feed ranged from 25.7–62.3 mg/kg. Chlortetracycline was among the most common additives in chicken and pig feed ( Van Cuong *et al.*, 2016). Thus it is not unusual to find both copper and tetracycline in the same environments.

## Conclusion

Preservation of antibiotics as useful medicines requires stewardship of populations of bacteria that remain susceptible to them. It is imprudent to base stewardship on frequency of resistance because even low numbers of resistant bacteria will dominate a population when antibiotics are used. Environments that maintain phenotypes caused by adaptive resistance or genotypes with a fitness advantage during antibiotic exposure thus could contribute to the rate at which populations of pathogens evolve resistance ( Kurenbach *et al.*, 2018).

The number of circulating high use commercial chemicals being associated with sub-lethal effects on bacteria is growing, as are the number of environments that are being contaminated with antibiotics themselves. Exposure to herbicides and antibiotics simultaneously accelerates the evolution of genotypically resistant bacteria ( Kurenbach *et al.*, 2018). The effects seen for atrazine, copper and pyrethrins were more limited than for some other herbicide active ingredients and commercial formulations, but may contribute to the overall burden of resistance.

## Data availability

### Underlying data

All underlying data is available on the Open Science Framework: Effects of sub-lethal concentrations of copper ammonium acetate, pyrethrins and atrazine on the response of Escherichia coli to antibiotics, https://doi.org/10.17605/OSF.IO/RZKWU ( Kurenbach, 2018).

The following files are available:

- **Effects of biocides on antibiotic response.**Antibiotic resistance in the presence and absence of biocide. Data presented in Figure 1.
  ○ Atrazine+Cip_killing curves.csv
  ○ Atrazine+Kan_killing curves.csv
  ○ Atrazine+Str_killing curve.csv
  ○ Atrazine+Tet_killing curve.csv
  ○ Cu+Cip_killing curves.csv
  ○ Cu+Kan_killing curves.csv
  ○ Cu+Str_killing curves.csv
  ○ Cu+Tet_killing curves.csv
  ○ Pyrethrins+Cip_killing curves.csv
  ○ Pyrethrins+Kan_killing curves.csv
  ○ Pyrethrins+Str_killing curves.csv
  ○ Pyrethrins+Tet_killing curves.csv
- **Minimum inducing concentration.** Data presented in Figure 2.
  ○ Cu+Str_Minimum inducing concentration.csv
  ○ Cu+Tet_Minimum inducing concentration.csv
  ○ Pyrethrins+Str_Minimum inducing concentration.csv
- **Dependence on the AcrAB-TolC efflux pump as evidence of an adaptive response**. Antibiotic resistance response in the presence and absence of copper. Data presented in Figure 3.
  ○ AcrA_Cu+Tet_killig curves.csv
  ○ AcrB_Cu+Tet_killig curves.csv
  ○ TolC_Cu+Tet_killig curves.csv
- ***tolC* was induced by copper**. Relative fluorescence data for BW21003(pHJ101) in the absence and presence of copper. Data presented in Figure 4.

Fluorescense_PtolC induction.csv

- **Copper directly reduced available tetracycline.** Data presented in Figure 5.
  ○ Chelation_all timepoints.csv
  ○ Chelation experiment_ANOVA tables.docx

Data are available under the terms of the Creative Commons Zero "No rights reserved" data waiver (CC0 1.0 Public domain dedication).
