# Supplementary material for: Effects of sub-lethal concentrations of copper ammonium acetate, pyrethrins and atrazine on the response of Escherichia coli to antibiotics
Source: F1000Res. 2019 Jan 9;8:32. [Version 1] doi: 10.12688/f1000research.17652.1 (PMC6381802; doi:10.12688/f1000research.17652.1)
Supplement: Peer review history

# Peer review: Referee response for version 1

**DOI:** 10.5256/f1000research.19303.r42814

**Published:** 2019-02-18

**Authors:** Piklu Roy Chowdhury

**Affiliations:** 1 ithree institute, University of Technology Sydney, Sydney, NSW, Australia

This study, one of a series of reports by the group, is on the impact of sub-lethal concentrations of biocides on the ability of *Escherichia coli* to resist three different antibiotics, commonly used in the treatment of human diseases. Presently, the primary focus of research on antibiotic resistance is either on surveillance of drug resistance genes or on characterizing mechanisms of drug resistance. Research on anthropogenic agents which co-select for drug resistance or equip bacteria to resist drugs by alternative mechanisms is underexplored. Data presented in the manuscript addresses this knowledge gap.

 Previously published experiments protocols and analytical pipelines were used to generate data in this study.  Significant findings are logically discussed, citing examples from available literature.

 Although I am not an expert in statistical analysis, the authors have used the standard methodology. The overall results on the statistical analyses were therefore easy to follow. Interpretation of results is convincing and supports conclusions presented.

 There is one typographic error in the manuscript.

 Page 5, right hand column first line: “pHJ01 transformands of BW25113…..”

 Please change “transformands” to “transformants”.

I have read this submission. I believe that I have an appropriate level of expertise to confirm that it is of an acceptable scientific standard.

# Peer review: Referee response for version 1

**DOI:** 10.5256/f1000research.19303.r42817

**Published:** 2019-02-01

**Authors:** Pal J. Johnsen

**Affiliations:** 1 Department of Pharmacy, Faculty of Health Sciences, University of Tromsø - The Arctic University of Norway, Tromsø, Norway

This manuscript addresses important questions on potential non-antibiotic drivers of antimicrobial resistance evolution. Jun et al test the co-selective abilities of widely used pesticides and provide data suggesting that in particular copper ammonium acetate may co-select for pre-existing tetracycline resistance in *E. coli*.

 Major points: 

 1. Relevance: The manuscript would benefit from a stronger contextualisation with respect to how relevant the co-selective effects reported are. The minimum copper biocide concentration causing a change in tetracycline response is reported to be 120microgramml- this is according to published data referred to in the Discussion in the upper range of what is measured in piglets- questioning the relevance of the findings. There is a huge literature on these issues - for example a study from Europe suggest that copper concentrations can be relatively high in soils, see for example (Heijerick DG1, Van Sprang PA, Van Hyfte AD.,2006 ^1^).  

 To this end, the experiments on both the effects on acrAB-TolC knock-outs and mScarlet tolC promoter- fusions are performed at a much higher concentration (450microgramml) - this needs justification beyond the stated NOEL. 

 The dose response data presented in Figs 1 and 2 for the Copper/tetracycline combinations strongly suggest that there are selective effects at much lower concentrations than the authors conservatively report. I fully support this approach considering the chosen methodology. However, I suspect that clear fitness effects may be seen at much lower doses of copper ammonium acetate. This could be shown by mixed competitions between WT and tolC knock-out mutants over a range of copper concentrations, similar to what was shown for sub-MIC selection of antibiotic resistance (Gullberg et al 2011 ^2^). I do however suspect that a simpler assay using relative growth rates as a proxy for relative fitness at lower concentrations than 120microgramml copper ammonium acetate would increase the sensitivity of identifying a “minimum co-selective” coper concentration.

 2. Non- inheritable resistance/tolerance as underlying mechanism for reduced tetracycline susceptibility: The reported patching experiments where it demonstrated that E. coli survival rely on simultaneous presence of copper were done at 35microgramml tetracycline. In the dose responses presented in Fig. 2 10microgramml is used. Given the large mutational target for efflux alteration- how can you so categorically rule out existing mutations with MICs lower than 35microgramml without presenting sequence data and or doing proper MIC assays of several colonies recovered on LB plates? 

 Other points:

- Table 3: the fold - change description does separate synergy/antagonism- the strep/copper combination is reported as 5 fold shift- but it has an opposite sign as compared to the 40 fold difference reported for copper/tet- this is a little confusing.
- Fig. 2: Why are the biocide concentrations here reported in ppm?- microgramml is used throughout the manuscript.
- Tetracycline MICs for acrAB-tolC mutants are not presented- this should be included.
- Figure 4: a less than 2- fold increase in expression is reported as significant. The use of single cells here takes stochastic variation in fluorescence into account and I do find the data solid but it would be good if more experimental details were provided. How many individual cells were measured, number of biological replicates ect- it is hard to extract from the deposited data (at least for this reviewer).

I have read this submission. I believe that I have an appropriate level of expertise to confirm that it is of an acceptable scientific standard, however I have significant reservations, as outlined above.

## References

[1] HeijerickDGVan SprangPAVan HyfteAD: Ambient copper concentrations in agricultural and natural European soils: an overview. *Environ Toxicol Chem*.2006;25(3) :858-64 1656617210.1897/04-671r.1

[2] GullbergECaoSBergOGIlbäckCSandegrenLHughesDAnderssonDI: Selection of resistant bacteria at very low antibiotic concentrations. *PLoS Pathog*.2011;7(7) : 10.1371/journal.ppat.1002158 e1002158 10.1371/journal.ppat.1002158 21811410PMC3141051

# Peer review: Referee response for version 1

**DOI:** 10.5256/f1000research.19303.r42816

**Published:** 2019-01-28

**Authors:** Xue-Xian Zhang

**Affiliations:** 1 Institute of Natural and Mathematical Sciences, Massey University, Auckland, New Zealand

This manuscript describes the effects of three commonly used agrochemicals (copper ammonium acetate, pyrethrins and atrazine) on bacterial resistance to antibiotics when they are present at sub-lethal concentrations. The data revealed significant antagonistic interactions between copper and the tetracycline antibiotic. It is particularly interesting that copper is capable of restoring the growth of *E. coli *cells when tetracycline was added at high concentrations that would normally cause full growth inhibition. Furthermore, the authors performed a series of hypothesis-driven experiments, and the results propose two underlying mechanisms: copper-induced expression of the AcrAB-TolC multidrug efflux system, and the chelation of tetracycline by copper. These results are highly significant, and I have no major reservations towards the experimental design and data interpretation. The few minor comments listed below are mostly on writings.

1. Figure 1: I am wondering if bacteria were inoculated immediately after the addition of copper (and antibiotics), and whether there was a specific order for the addition of copper and antibiotics. I think this information is important and it should be provided in the Methods. This is because the toxic effects of copper are exerted by free Cu ions, not directly by copper ammonium acetate. There will be a process of copper ion releasing, and subsequent binding with other organic compounds present in the LB medium. We would thus expect different results when bacteria were inoculated immediately or left for some time after copper addition.
2. Figure 2: ppm is used here, but the results are discussed in the main text using ug/ml. It would be helpful to use the same concentration unit. Also, it is difficult to distinguish the two types of grey bars, so the authors should consider changing one of them into an empty bar.
3. Table 2: There was a two-fold difference of copper MICs between the *acrA/acrB* and *tolC* mutants, which has been overlooked in both the Results and Discussion. The AcrAB-TolC system is supposed to be induced by copper, and confers resistance to tetracycline (but not copper). Thus, it needs an explanation why inactivation of *acrAB-tolC* caused a reduction of MIC to copper, and furthermore, why there was a difference among the three mutants. For data presented in Figure 3 on copper plus tetracycline, the difference among three mutants has been noted but not discussed. A triple mutant of *acrA, acrB* and *tolC* may help further understand the mechanisms.
4. Figure 5: I like this experiment and would be interested in what the results will be if the authors have set up an additional control with the addition of copper only (tetracycline added at the time of inoculation). As mentioned above, copper ions released from copper ammonium acetate can potentially react with organic compounds in the medium. Thus, copper-tetracycline chelation is just one of the plausible explanations for the obtained data. It will certainly help improve our understanding if the authors had determined the dynamic change of copper ion concentrations in this experiment.
5. The current “discussion” is a little bit weak, given that many interesting points need to be specifically addressed in this work, e.g., the roles of the AcrAB-TolC system and the genotypic versus phenotypic adaptation. 

I have read this submission. I believe that I have an appropriate level of expertise to confirm that it is of an acceptable scientific standard.
